# Mechanisms of Post-Stroke Fatigue: A Follow-Up From the Third Stroke Recovery and Rehabilitation Roundtable

**DOI:** 10.1177/15459683231219266

**Published:** 2023-12-29

**Authors:** Annapoorna Kuppuswamy, Sandra Billinger, Kirsten G. Coupland, Coralie English, Mansur A. Kutlubaev, Lorimer Moseley, Quentin J. Pittman, Dawn B. Simpson, Brad A. Sutherland, Connie Wong, Dale Corbett

**Affiliations:** 1 Queen Square Institute of Neurology, University College London, London, UK; 2 Department of Biomedical Sciences, University of Leeds, Leeds, UK; 3 Department of Neurology, University of Kansas Medical Center, University of Kansas Alzheimer’s Disease Research Center, Fairway, KS, MO, USA; 4 School of Biomedical Sciences and Pharmacy, College of Health, Medicine and Wellbeing, University of Newcastle, Australia Heart and Stroke Program, Hunter Medical Research Institute, Newcastle, NSW, Australia; 5 School of Health Sciences, College of Health, Medicine and Wellbeing, University of Newcastle, Australia Heart and Stroke Program, Hunter Medical Research Institute, Newcastle, NSW, Australia; 6 Department of Neurology, Bashkir State Medical University, Ufa, Russia; 7 IIMPACT in Health, University of South Australia, Adelaide, SA, Australia; 8 Department of Physiology and Pharmacology, Hotchkiss Brain Institute, University of Calgary, Calgary, AB, Canada; 9 School of Health Sciences, College of Health, Medicine and Wellbeing, University of Newcastle, Australia Heart and Stroke Program, Hunter Medical Research Institute, Newcastle, NSW, Australia; 10 Tasmanian School of Medicine, College of Health and Medicine, University of Tasmania, Hobart, TS, Australia; 11 Centre for Inflammatory Diseases, Department of Medicine, School of Clinical Sciences at Monash Health, Monash University, Clayton, VIC, Australia; 12 Department of Cellular and Molecular Medicine, University of Ottawa Brain and Mind Institute, University of Ottawa, Ottawa, ON, Canada

**Keywords:** post-stroke fatigue, mechanisms, inflammation, neural networks, exercise, dopamine

## Abstract

**Background:**

Post-stroke fatigue (PSF) is a significant and highly prevalent symptom, whose mechanisms are poorly understood. The third Stroke Recovery and Rehabilitation Roundtable paper on PSF focussed primarily on defining and measuring PSF while mechanisms were briefly discussed. This companion paper to the main paper is aimed at elaborating possible mechanisms of PSF.

**Methods:**

This paper reviews the available evidence that potentially explains the pathophysiology of PSF and draws parallels from fatigue literature in other conditions. We start by proposing a case for phenotyping PSF based on structural, functional, and behavioral characteristics of PSF. This is followed by discussion of a potentially significant role of early inflammation in the development of fatigue, specifically the impact of low-grade inflammation and its long-term systemic effects resulting in PSF. Of the many neurotransmitter systems in the brain, the dopaminergic systems have the most evidence for a role in PSF, along with a role in sensorimotor processing. Sensorimotor neural network dynamics are compromised as highlighted by evidence from both neurostimulation and neuromodulation studies. The double-edged sword effect of exercise on PSF provides further insight into how PSF might emerge and the importance of carefully titrating interventional paradigms.

**Conclusion:**

The paper concludes by synthesizing the presented evidence into a unifying model of fatigue which distinguishes between factors that pre-dispose, precipitate, and perpetuate PSF. This framework will help guide new research into the biological mechanisms of PSF which is a necessary prerequisite for developing treatments to mitigate the debilitating effects of post-stroke fatigue.

## Introduction

Post-stroke fatigue (PSF) is a significant symptom for stroke survivors with few effective, evidence-based interventions currently available. The lack of evidence-based interventions is largely a result of poor understanding of the phenomenon, with little agreement on its definition and measurement. Fatigue is a complex phenomenon with multiple driving factors, which requires a systematic deconstruction of the phenomenon to propel advances in the field. This aim was pursued following the recent Stroke Recovery and Rehabilitation Roundtable (SRRR) consensus process involving experts in the field, which has produced a comprehensive definition and guidelines for measurement of PSF alongside a brief exposition on the possible mechanisms of fatigue and available interventions.^1^

In this companion SRRR paper we have put forward a clear definition of fatigue that incorporated both expert consensus and personal experience of stroke survivors. PSF is not mere tiredness, but a “feeling of exhaustion, weariness or lack of energy that can be overwhelming, and which can involve physical, emotional, cognitive and perceptual contributors, which is not relieved by rest and affects a person’s daily life.” Previous studies of PSF have frequently been confounded by other conditions such as depression, anxiety, and sleep disorders which often associate with fatigue.^2^ While these conditions might contribute to the feeling of fatigue, they are dissociable and need to be identified at the time of diagnosis. For example, Fluoxetine relieves depression but not fatigue.^3^ The consensus view of our SRRR working group was that the Fatigue Severity Scale-7 (FSS-7) represented the most commonly used fatigue measure. Despite its wide usage, this scale has several drawbacks as it does not distinguish between different domains and does not measure fatigue severity or the impact of fatigue on communication ability. It primarily captures impact and interference of fatigue in daily life. For research purposes nuanced interpretations of findings will require the use of domain specific scores from other elaborate fatigue scales summarized in the main paper.^1^ Clinically, in order to ensure that PSF does not continue to be an invisible symptom, it is important that it is detected as soon as possible following stroke. We have recommended that the Stroke Fatigue Clinical Assessment Tool (SF-CAT) best meets this need. The SF-CAT can be administered via interview and should be part of clinical follow up for all stroke survivors.

The primary goal of the current current paper is to elaborate on mechanisms of PSF briefly discussed in a companion SRRR paper on PSF.^1^ Here, we present a more comprehensive description of the potential processes that drive PSF in order to guide future research into the biological mechanisms of PSF and ultimately the development of new therapeutic interventions. We draw from the literature both in stroke and other diseases where fatigue is a significant symptom and put forward a model of PSF that further highlights promising avenues of future research.

**Figure 1.**
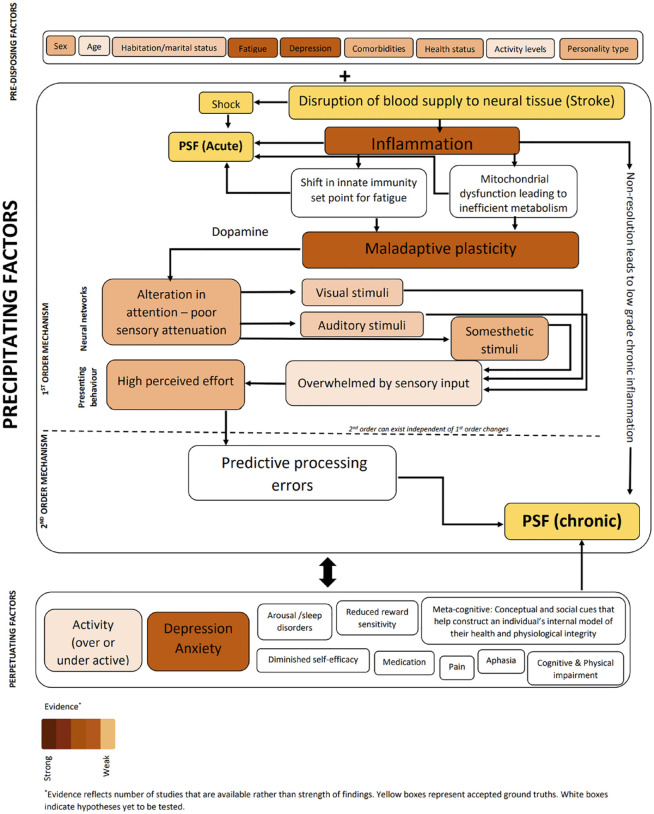
Unifying model of fatigue. This schematic illustrates factors that are associated with PSF and its potential role in development and maintenance of PSF. New and untested hypotheses previously proposed, are also included.

We begin by presenting the idea of PSF as a cluster of disorders with potentially dissociable mechanisms. We then discuss evidence that supports inflammation and immune dysregulation as a potential process that could underpin both acute PSF and long-term PSF. Next, we discuss how dopamine (DA), a neuromodulator with diverse functions including effort perception, motivation, and memory, could be implicated in PSF, with evidence supporting dopaminergic pathways as a potential therapeutic target. Finally, we discuss whole brain neural network changes and exercise induced multi-system dynamics in the context of PSF, both mechanistically and therapeutically. Furthermore, throughout the manuscript, we present evidence from other human diseases where fatigue is a significant symptom, to identify possible overlapping mechanisms with PSF. This is based on the premise that fatigue, in the chronic stages of a disease is delinked from the primary etiopathology of the disease and commonalities in the experience of chronic fatigue indicate a common disease-independent mechanism. Finally, we present a single framework (Figure 1) that links the available evidence and identifies the gaps in our knowledge about PSF.

## PSF: Single or Multiple Disorders?

A fundamental question is whether PSF is a uniform disorder or 1 with distinct subtypes. At an individual level, evidence from qualitative studies suggests that PSF is experienced differently, with each individual experiencing 1 or more predominant features of PSF. Such diversity of experiences alongside comorbidities in PSF, suggests there may be different subtypes that warrant phenotyping. Some stroke survivors describe PSF as physical activity requiring more effort while others define it as an inability to follow conversations, the perception of brain fog, or frequent occurrence of emotional outbursts.^4^ Likewise, triggers for fatigue also vary, which results in adoption of different coping strategies. Some stroke survivors take short rest periods to relieve fatigue, others engage in physical activity to relieve fatigue while others find cognitive task switching an effective strategy.^5^ There is no comprehensive study investigating a systematic relationship between triggers, experience, and coping mechanisms related to PSF. But the report of distinct features, triggers, and effective coping methods that are specific to the individual suggest there may be subtypes of PSF.

Further evidence for the potential for subtypes of fatigue comes from quantitative studies. The majority of PSF studies use the FSS which provides a single score for fatigue. Other instruments such as Modified Fatigue Impact Scale (MFIS) and Fatigue Scale for Motor and Cognitive (FSMC) functions have subscale scores. Each scale measures different aspects of fatigue and recent recommendations by the SRRR roundtable highlights the need for carefully choosing the scale to suit the aspect of fatigue being interrogated, which will allow for easy cross comparison across investigations.^6^ Studies that have used scales with sub-scores show that physical impairments are associated with higher scores in the physical subscale of MFIS, while cognitive impairments relate to mental subscale score of MFIS.^7^ Cognitive subscale scores of FSMC related to mental speed, working memory, and verbal short-term memory, while the physical subscale additionally correlated with upper limb physical function. Moreover, those with cortical lesions scored higher on cognitive subscale scores, and those with subcortical lesions scored higher on physical subscale scores.^8^ Binocular visual dysfunction relates to both physical and cognitive fatigue, while gait disturbances are associated with cognitive fatigue.^9^ Studies that measured only mental fatigue, showed a relation with several measures of cognitive impairment.^10^ Those with depression are more likely to score high on the mental and motivational subscales, while physical fatigue scores are more strongly influenced by anxiety.^11^ Overall, when measured using an instrument that distinguishes the dimensions of PSF, the correlation with functional measures is dissociable, suggesting that different underlying pathologies might manifest as different aspects of the experience of fatigue.

In stroke patients in the late subacute (>3 months) or chronic stroke phases (>6 months) Modafinil is effective^12^ for PSF, but only in those with fronto-striatal dysfunction.^13^ In a similar population, neuromodulation interventions^14,15^ show mixed results, due to differences in methodologies, with bilateral cortical sensorimotor targets providing robust reduction in fatigue, and unilateral dorsolateral pre-frontal targets showing no effects. Furthermore, participants in the sensorimotor cohort were minimally impaired with no depression, while in the dorsolateral pre-frontal cohort although minimally impaired, depressed individuals were not excluded. Therefore, mixed results could be due to both participant and intervention characteristics as suggested by the effectiveness of both graded exercise and cognitive behavioral therapy, but only in a small subset of stroke survivors.^16^ The selective effectiveness of different types of interventions suggests the presence of subtypes, each of which may respond to a different intervention.

PSF significantly overlaps with depression, anxiety, pain, and sleep disturbances^17-19^ in stroke survivors. While the general consensus is that the primary underlying mechanisms of fatigue are independent of comorbidities, treatment strategies may need to accommodate for the presence of a significant comorbidity, or indeed there may be overlapping mechanisms which need further investigation, necessitating comorbidities-based phenotyping.

## Precipitating Factors of PSF

Current evidence supporting a role for inflammation contributing to PSF is limited but evidence suggests that the acute inflammatory response may persist longer than previously thought.^20-22^ Small studies aimed at identifying inflammatory biomarkers of PSF, have made assessments early after stroke rather than later in the subacute or chronic (>6 months) post-stroke phase. Despite this, a positive correlation between acute interleukin-1β (IL-1β; a key mediator of the inflammation response) levels and fatigue 6 months after stroke has been observed (*r* = .37), whereas anti-inflammatory IL-1ra (*r* = −.38) and IL-9 (*r* = −.36) levels negatively correlated with fatigue at 12 months.^23,24^ The relationship between levels of C-reactive protein (CRP) and fatigue are unclear with 1 study showing a positive relationship (odds ratio 3.435, 95% confidence interval 2.222-5.309), another a negative relationship (ρ = −.47, *p* < .05) and a further study finding no relationship (*r* = .12).^25-27^ However, a recent retrospective study using a high sensitivity test for CRP showed that PSF at 6 months after stroke was associated with increases in CRP at admission, indicative of systemic inflammation.^28^

Stroke-induced inflammation may also lead to genomic or epigenetic changes which modulate brain energy metabolism.^25^ The brain has limited energy supplies which are further taxed by brain injury and advancing age as additional neural networks are recruited to maintain function. Systemic inflammation can disturb mitochondrial function, shifting it towards a more inefficient form of metabolism; this phenomenon has also been observed in cancer and chronic fatigue syndrome.^29^ Thus, it is highly possible that mitochondria may have a smaller “reserve energy capacity” after stroke, making them unable to provide sufficient ATP to meet the energy demand. This bioenergetics hypothesis may explain variation in PSF amongst patients as they respond differently to external/internal factors, such as sleep changes, stress level, and changes in co-morbidity. Microglia, the resident immune cells of the brain, become “primed” after brain injury, resulting in prolonged activation and chronic neuroinflammation. In traumatic brain injury, persistent microglial activity or inflammation promote neuronal dysfunction and impairments in neuronal homeostasis.^30,31^ Perhaps similar perturbations occur after stroke and contribute to neuropathology in PSF. Excessive cytokine production and immune dysregulation could also have an impact on orexin-secreting neurons in the hypothalamus as well as a decreased synthesis of serotonin, DA, and norepinephrine neurotransmitters.^32^ Other studies show that a loss of activity in these orexin neurons could lead to fatigue.^33^ While no investigations so far have directly investigated hypothalamic–pituitary–adrenal (HPA) axis dysfunction in PSF, there is a high prevalence of pituitary dysfunction in stroke^34^ and warrants a thorough investigation to determine if this could be related to inflammation. In a cross-sectional study (n = 70) of participants in the chronic phase of stroke recovery, fatigue score was significantly positively correlated with the level of both IL-6 and CRP, although this relationship was no longer statistically significant once entered into a linear regression model with cardiovascular covariables.^20^ Therefore, PSF may not be a direct result of inflammation, but may be driven by a matrix of other factors such as the stroke associated cardiovascular risk factors (e.g. diabetes, hypertension). There are also genetic factors that might predispose patients to PSF as has been reported for single-nucleotide polymorphisms in genes that modulate inflammation. One study reported that PSF is associated with the C allele of IL1RN rs4251961. In addition, functional polymorphisms in the gene for toll-like receptor 4 (*TLR4*) that render TLR4 less responsive to its ligands are associated with lower levels of PSF.^35^ Given the limited research to date, it is apparent that definitive inflammatory biomarkers for PSF remain to be identified. However, the data overall support the idea that perturbations in the immune response after stroke may contribute to PSF.

Another possible contributor to PSF is post-stroke dysbiosis, which has a bidirectional relationship with host immune and inflammatory response following injury. The effects of a post-stroke inflammatory state on gut dysbiosis as a driver for PSF has not been examined but warrants investigation, especially in light of emerging evidence in support of inflammation induced gut dysbiosis as a possible driver of fatigue in other conditions such as cancer related fatigue,^36^ and the beneficial effects of fecal transplantation in stroke.^37^ If dysregulation of the immune response is an important factor in PSF, then an appropriate treatment strategy may be to dampen the inflammatory response, similar to work in Multiple Sclerosis. However, the exact targets need to be better elucidated given the complex network of signaling mechanisms controlling inflammation, and the complexity around the development of PSF.

DA neurons are involved in the control of movement, motivation, arousal, learning, and regulation of the immune system.^38^ There is evidence that damage to DA pathways is associated with fatigue in multiple sclerosis.^39^ Further, the DA reuptake inhibitors, methylphenidate and modafinil have been used to treat PSF.^12,40,41^ Methylphenidate reportedly reduces fatigue in small studies of chronic fatigue,^41^ Multiple Sclerosis, Parkinson’s disease, and cancer.^39,42^ There is evidence that methylphenidate reduces the cost of mental labor (ie, perceived effort). Participants given methylphenidate engage in challenging cognitive tasks more often than leisure activity when compared to placebo controls.^43^ [18F] DOPA positron emission tomography (PET) imaging showed that this effect was associated with higher striatal DA synthesis. Methylphenidate’s effectiveness in PSF has not been assessed. Modafinil shares many actions with methylphenidate and may offer some benefit in reducing PSF as reported in 2 small trials.^12,44^ In the MIDAS trial, positive responders to modafinil treatment had previously exhibited reduced functional connectivity in the fronto-striatal-thalamic networks when compared to those who did not respond to modafinil.^13^ The selective effectiveness of Modafinil in this study suggests that more than 1 mechanism might underlie PSF and non-responders might require a different intervention for reduction of PSF. A larger, multi-center clinical trial (MIDAS 2) investigating the capacity of modafinil to improve stroke patient quality of life and reduce fatigue, is underway and will throw further light on characteristics of individuals in whose PSF is reduced by modafinil.

The pharmacological evidence linking DA to PSF is thus based upon the efficacy of modafinil and methylphenidate to increase DA concentration by inhibiting its uptake. In that scenario, the DA hypothesis of fatigue is similar to the serotonin hypothesis of depression which is based upon the efficacy of the selective serotonin reuptake inhibitors (eg, Fluoxetine). Going forward, trials should include functional magnetic resonance imaging or PET imaging that could be used to relate behavioral outcomes to changes in functional network connectivity, including DA activity. An important caveat for future studies is that combination therapies are much more effective than single interventions in improving cognition, and post-stroke motor recovery.^45,46^ For example, preclinical and clinical studies indicate that a combination of physical exercise and cognitive stimulation are more effective than either alone in improving cognition. It may be that DA reuptake inhibitors need to be combined with other interventions such as exercise or cognitive training^16^ to achieve more robust reductions in PSF. This would be in line with the view that PSF consists of both *peripheral* (eg, disability due to stroke and physical deconditioning) and *central* fatigue.

Over the last 2 decades several small studies (see review^47^) have investigated the relationship between lesion characteristics and PSF with inconclusive results partly due to the variations in scales, metrics of lesion characteristics, small sample size, and variations in time points. Recently, a meta-analysis^48^ of 14 studies assessing PSF, and a large scale (n = 361) structural MRI study^49^ both concluded that a lesion in the thalamus significantly increased the likelihood of reporting PSF 6 months post-stroke. Extending beyond lesion location, another study examined if structural dysconnectivity associated with the lesion explains PSF, and found no relationship.^50^ The thalamus is a major sensory processing hub with functionally heterogenous nuclei and several first and second order processing units. Current studies do not differentiate between different thalamic nuclei and networks. For lesion mapping studies to meaningfully contribute to understanding the mechanisms of PSF, it is critical that future studies use fine grained methods of lesion mapping which combines both structural and functional outcomes,^51^ along with use of PSF instruments that differentiate the dimensions of PSF. Such an approach may also yield better outcomes for structural and functional connectivity mapping of PSF. An equally potent, alternate method would be to combine clinically available lesion-mapping data with longitudinal follow-up of PSF associated behavior to identify functionally relevant networks involved in PSF.

Fatigue is an experience, and experiences are an emergent property of neural network activity. Investigations that have focused on neural network activity in PSF have explored the possibility of network level dysfunction as the basis of PSF. A recently proposed model, the sensory attenuation model of fatigue suggests that altered gain modulation in sensory networks, results in PSF.^52,53^ The theory posits that task irrelevant sensory information, which is normally attenuated, is poorly suppressed leading to high task-related effort. Fatigue is an inference of high effort; therefore, poor sensory attenuation mechanistically underpins fatigue. Biomarkers of poor attenuation include changes in resting state cortical excitability and neural network connectivity, all of which are present in PSF.^54,55^ An inability to suppress muscle contraction related afferent input manifests as resting state hyper-connectivity in primary somatosensory networks, and hypoconnectivity in motor networks in PSF.^55^ Evoked potentials from M1 are diminished in PSF^54^ further corroborating heightened sensory processing which has inhibitory influences on M1 excitability. Such altered resting state of sensorimotor neural networks has implications for brain states that need to be achieved in order to produce a movement. In PSF, poor modulation of pre-movement motor cortical inhibition^56^ is observed which is a hallmark of poor behavioral flexibility underpinned by heightened sensory processing. Sensory processing is also reliant on inter-hemispheric interactions, and the normal left hemispheric dominance in such interactions shifts to a right hemispheric dominance^57^ in people with PSF. Attention to visual and auditory sensory streams also appear to be poorly attenuated, specifically in relation to processing of distractors both in auditory^58^ and visual processing^59^ in PSF. This series of results support the sensory attenuation model of fatigue, however, functional connectivity studies beyond the somatosensory-motor networks do not fully support the hypothesis,^60^ but indicate network level dysfunction does underpin PSF. Evidence from other diseases such as multiple sclerosis^61,62^ also supports a neural network dysfunction hypothesis of fatigue, with white matter lesions implicated in network dysfunction. In stroke, despite no white matter involvement, small vessel disease is independently associated with greater fatigue.^63^ Neuromodulation of sensorimotor networks significantly reduces fatigue both in stroke^14,64^ and in multiple sclerosis^65,66^ suggesting a causal link between sensorimotor dysfunction and fatigue. While more studies are needed to replicate and expand the observed results, neural network dysfunction is a possible driver of PSF and a promising interventional target. Neural networks do not function in isolation and, the impact and interaction of sensorimotor network dysfunction with other networks in relation to fatigue is unknown. Lack of studies focusing on pre-frontal and sub-cortical networks that subserve movement, attention, and motivation makes it difficult to definitively conclude the importance of sensorimotor dysfunction over others, for development of PSF. Future research must focus on exploring these other network level dysfunctions in PSF.

## Exercise as a Possible Intervention for PSF

People with stroke are much less active than age-matched controls.^67,68^ Exercise may ameliorate some of the peripheral (aerobic deconditioning and muscle atrophy) and central disturbances (alterations in cerebral blood flow, cellular energy stores, and neural circuit activity) that potentially contribute to PSF.^36-71^ In a small RCT using a combination of 12-weeks of cognitive training and exercise, addition of exercise reduced fatigue compared to control subjects that received cognitive training alone.^16^ A recent systematic review explored whether exercise impacts fatigue using the FSS.^72^ Only 2 of 4930 manuscripts met the defined eligibility criteria. Regan et al^73^ used a 9 to 10 weeks combination of aerobic and resistance training that significantly reduced PSF immediately following the intervention while the effect was lost 20 weeks post-intervention. Paul et al^74^ also reported significant reduction in PSF following a 6-week aerobic exercise program consisting of increasing steps. Both studies suffered from small sample size, lack of details about stroke (type and location), and/or details of the exercise programs (frequency and session duration) with the additional lack of a control arm in the first study, and fatigue not the primary target of intervention in the second study. Other conditions which exhibit fatigue symptomology have also shown benefit with exercise including multiple sclerosis,^75,76^ cancer, chronic fatigue syndrome, and other diseases.^77,78^ For example, a randomized clinical trial reported that a 16-week combination of resistance training and high intensity interval training reduced fatigue in breast cancer patients.^78^

Given that exercise increases aerobic conditioning, cerebral blood flow, release of growth factors (eg, BDNF), DA, and other monoamine neurotransmitters, neurogenesis, and decreases inflammation,^71^ it is surprising that it has received so little attention as a potential treatment for PSF. While there are several pathways through which exercise could reduce fatigue, only recently have investigations in disease populations begun to investigate these pathways. For example, in Multiple Sclerosis, exercise is thought to reduce fatigue by reducing the levels of the muscle derived hormone, irisin.^79^ Aerobic exercise alone, or in combination with resistance training or cognitive training, may be helpful in reducing PSF (keeping in mind that the “doses of rehabilitation” have typically been too low to engage neuroplasticity mechanisms important for post-stroke motor recovery).^80,81^ Careful titration of dose will be required in PSF patients who are either deconditioned and/or exercise intolerant. Another potentially important consideration for exercise-PSF studies is timing. As is the case with motor recovery studies, most exercise-PSF trials have been started in the chronic phase—2 or more years post-stroke. Delaying motor rehabilitation into the chronic phase makes it progressively more difficult to achieve significant recovery.^46,82^ PSF may benefit from interventions implemented earlier to prevent deconditioning and to preserve energy expenditures that may be limited by supply. An important consideration is that people with a history of fatigue may become resigned to their condition and resistant to engage in exercise that they perceive as potentially harmful. Interventions to mitigate against this should be explored and if it remains an insurmountable problem exercise mimetic, such as drugs that activate many of the same cellular signaling pathways as aerobic exercise^83^ might be helpful as adjunctive therapies to reduce PSF. These drugs could be especially helpful in patients who are severely deconditioned or fearful of engaging in exercise.

## A Unifying Model of Fatigue

Stroke sets into action a complex cascade of events that spans multiple neural control systems, with significant impact on several peripheral and central pathways. Fatigue is a complex phenomenon often not explained by any 1 dysfunctional biological process resulting from stroke. In what follows, we attempt to bring together known factors that underpin fatigue as discussed in previous sections, and present a framework that will facilitate interpretation of results, and development of effective interventions (Figure 1). In this model, we follow a recently proposed principle of segregating factors associated with complex symptoms into that which pre-disposes one to experience fatigue, factors that are essential for experience of fatigue (precipitating factors), and those that perpetuate fatigue.^84^ While outside the scope of this paper, there are several known pre-disposing factors associated with PSF^19,85^ which are included in the model, and discussed in the SRRR paper. The essential conditions for development of PSF start with high levels of inflammation setting into motion changes in innate immune response and changes in metabolic pathways. Stroke profoundly disrupts neurotransmission, with specific emphasis on DA signaling. The impact of dopaminergic dys-homeostasis is reflected in whole brain networks, specifically sensory networks processing somesthetic, visual, and auditory stimuli, where gain modulation, a process dependent on DA, is compromised. Such compromise in sensory processing can present independently or as a cluster giving rise to the rich range of symptoms reported by those with PSF. Over time, with the resolution of stroke related high levels of inflammation, second order mechanisms such as alterations in predictive processing triggered by altered sensory gain, establishes itself as the primary driver of chronic fatigue. The interplay between precipitating factors and a range of perpetuating factors such as comorbid depression, pain, sleep disorders, anxiety, psychosocial factors, all result in exacerbation and maintenance of fatigue in the long-term.

To reverse fatigue the precipitating factors must first be addressed. While presently aspirational, targeted, hypothesis driven research to establish the key nodes of fatigue related dysfunction is critical in developing effective interventions. Effective interventions will likely be implemented early in the post-stroke period and entail a combination of pharmacological, neuromodulatory, and behavioral, interventions that address mechanisms at several levels. An opportunity to modulate fatigue levels presents itself in the perpetuating factors where a number of paradigms are already in trial, or in existing clinical practice. Such paradigms need to be rigorously tested and best practice widely disseminated to optimize results for stroke survivors.

## Conclusion

In summary, PSF is a complex phenomenon which requires a multi-disciplinary approach that cuts across clinical and basic science disciplines for a comprehensive understanding. The ball has been set rolling by the SRRR roundtable with a comprehensive definition, guidelines for how to measure PSF and in the present paper an in-depth discussion of the biological correlates of PSF. We also identify promising avenues for future research and highlight the need for a hypothesis driven approach in future mechanistic investigations of PSF.
